# A Rare Case of Ibuprofen-induced Acute Liver Injury

**DOI:** 10.7759/cureus.3225

**Published:** 2018-08-29

**Authors:** Vahe Shahnazarian, Daryl Ramai, Madhavi Reddy

**Affiliations:** 1 Gastroenterology and Hepatology, The Brooklyn Hospital Center, Affiliate of the Mount Sinai Hospital, New York, USA; 2 Internal Medicine, The Brooklyn Hospital Center, Affiliate of the Mount Sinai Hospital, New York, USA; 3 Gastroenterology and Hepatology, The Brooklyn Hospital Center, Affiliate of the Icahn School of Medicine at Mount Sinai, New York, USA

**Keywords:** hepatology, liver, nsaid

## Abstract

Idiosyncratic drug-induced liver injury (iDILI) is the second-most-common cause of acute liver injury. When it is caused by ibuprofen, it is quite rare, especially when not accompanied by systemic signs or symptoms. A young female patient presented with an ibuprofen overdose suicide attempt and then developed an acute liver injury within a few days. Given its rarity, ibuprofen-induced iDILI was initially a secondary differential, but when her course did not improve as expected, she was quickly evaluated for liver transplant. She fully recovered without needing the transplant, but this case highlights the importance of not only early suspicion/detection but also early referral to a transplant hepatology service.

## Introduction

Cases of iDILI due to ibuprofen are quite rare, occurring in approximately one per 100,000 patients. Even then, the patient will typically have a severe hypersensitivity reaction associated with the liver injury, such as toxic epidermal necrolysis or Stevens-Johnson syndrome [1].

While it is known that chronic ibuprofen use may cause an elevation in transaminases; the elevation is typically no more than the low 100s. An ibuprofen overdose (greater than 5-10 grams) is typically associated with altered mental status, respiratory depression, coma, and lactic acidosis, which may prove fatal. Most cases have not been associated with liver injury [1].

The purpose of this case report is to illustrate that, while very rare, an ibuprofen overdose may result in an acute liver failure. It is important to keep this in mind because a rapid diagnosis will result in the earlier involvement of a transplant hepatology team in cases of fulminant liver failures.

## Case presentation

A 29-year-old Caucasian female was brought to the emergency department (ED) in the late afternoon by ambulance for altered mental status. Earlier that day, her mother had gone to her apartment, at which time, the patient had become more confused and lethargic, prompting the phone call to emergency services. In the ED, she was lethargic and not answering questions. As per her mother, she had confessed to taking a large bottle of ibuprofen in a suicide attempt earlier that morning. In all, she had taken approximately 300 tablets of 200 mg ibuprofen (approximately 60,000 mg in total). Of note, she had no known allergies to medications. She had a medical history, including depression, asthma, alcohol abuse, and prior drug abuse (cocaine, Percocet, and intravenous heroin). In fact, she had completed a drug rehabilitation program six months ago and had not been drinking or using illegal drugs since then. She still smoked half a pack of cigarettes per day “for years” and would occasionally have an alcoholic beverage with friends. Her surgical history included breast reduction surgery. Her father had a history of hypertension, her mother had non-alcoholic fatty liver disease (NAFLD), and her aunt (mother’s sister) had cryptogenic cirrhosis).

Her vital signs in the ED were a temperature of 98 degrees Fahrenheit, a pulse of 111 beats per minute, a blood pressure of 109/66 mmHg, a respiratory rate of 17, and an oxygen saturation of 97% on room air. Her physical exam was non-revealing other than her lethargy. Her initial complete blood count (CBC) and basic metabolic panel (BMP) were within normal limits. She was intubated for airway protection and was admitted to the medical intensive care unit (MICU) for further treatment. She was then emergently hemodialyzed overnight for ibuprofen overdose (early morning of Day 2). On the morning of Day 3, her mental status had returned to her normal baseline, so she was successfully extubated. On Day 4, the MICU team noted an elevation in her liver enzymes (LFTs). From a baseline of normal, her total bilirubin was now 2 mg/dL, aspartate aminotransferase (AST) 350 U/L, alanine aminotransferase (ALT) 383 U/L, albumin 2.3 g/dL, and international normalized ratio (INR) was 1.5. Her alkaline phosphatase (ALP) was normal. Gastroenterology was consulted at that time. She denied any history of liver disease. She stated she had not recently taken any supplements, vitamins, over-the-counter medications (other than the ibuprofen), herbal medications, or herbal teas. She denied any abdominal pain, nausea, vomiting, hematemesis, melena, or hematochezia. Vital signs were within normal limits and stable. On exam, she appeared obese, jaundiced, and had multiple tattoos on her body. She had no abdominal tenderness to palpation, no appreciable abdominal organomegaly, and had appropriate bowel sounds. She was thought to have had transaminitis due to a possible ischemic liver from her initial borderline hypotension. Ibuprofen toxicity was also considered but as a secondary differential given its rarity. Recommendations were made to start the patient on N-acetyl cysteine (NAC), trend her LFTs, and start a daily proton pump inhibitor by mouth, to obtain a right upper quadrant sonogram, to rule out other causes of hepatitis, including viral and autoimmune, and to contact the local transplant hepatology service to discuss the patient.

**Figure 1:**
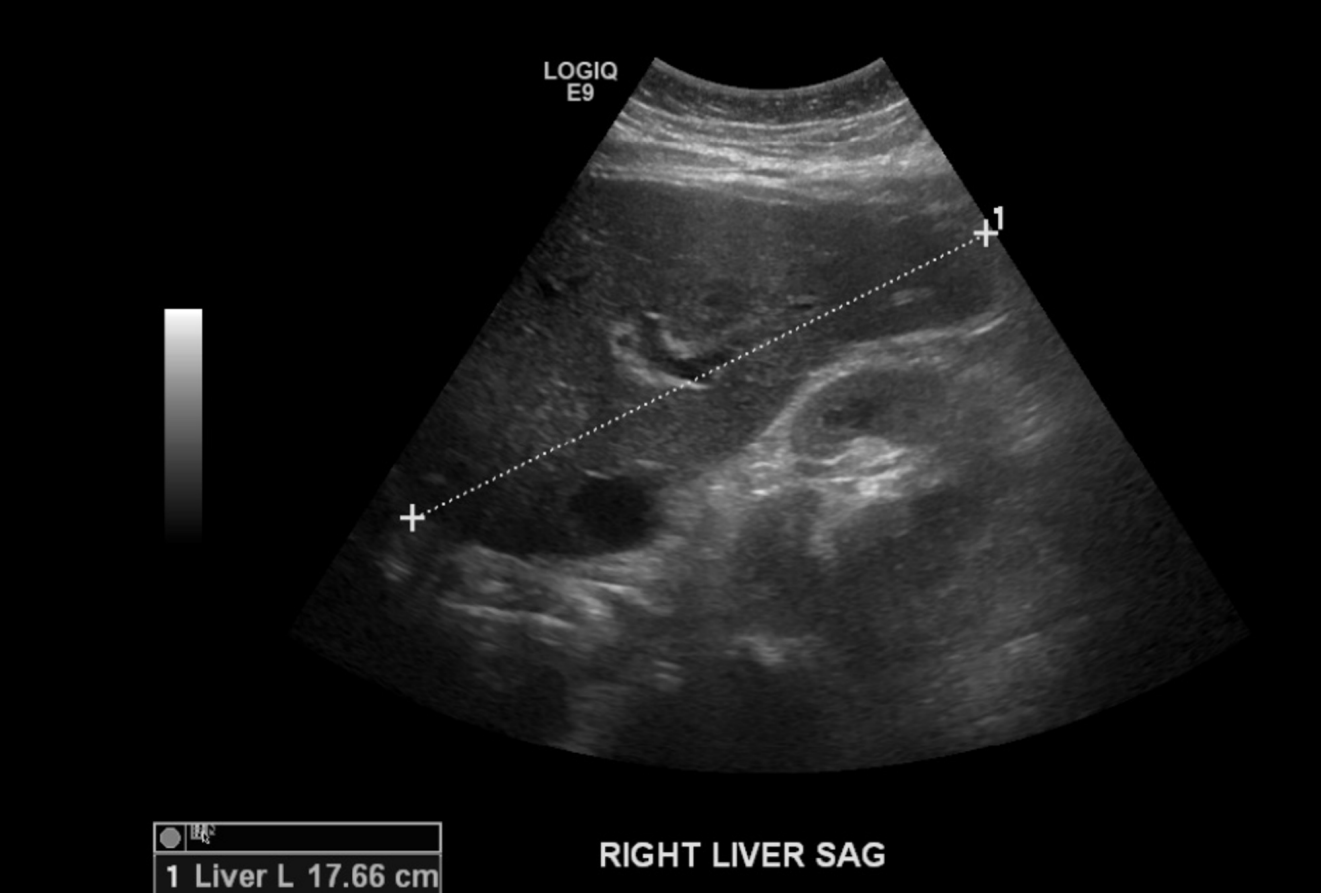
Enlarged liver on sonogram

On Day 5, her LFTs continued to trend up. She tested immune to hepatitis A and B and negative for hepatitis C and HIV. Her ferritin was elevated to 1664 ng/mL, anti-nuclear antibody was positive (ratio of 1:18), anti-mitochondrial antibody was negative, anti-smooth muscle antibody was positive, herpes simplex 1 and 2 were both positive, cytomegalovirus antibody was positive, varicella zoster virus was negative, Epstein-Barr virus antibody was positive, alpha 1-antitrypsin was negative, and ceruloplasmin was negative. The sonogram showed an enlarged liver (17.7 cm), hypoechoic in texture and indicative of hepatitis (Figure 1). The transplant hepatology service stated that she was not a candidate for emergent liver transplant at this time, given her appropriate mental status. On Day 6, her LFTs continued to trend up and she began to have intermittent, watery diarrhea, occasionally streaked with bright red blood. Her hemoglobin stayed within the normal range and she tested negative for Clostridium difficile. She was also given a total of 15 mg of vitamin K for her elevated INR of 1.9. On day 7, her LFTs peaked, with a total bilirubin of 5mg/dL, AST > 717U/L, ALT 1873U/L, and albumin 2.5g/dL. Her platelets had also steadily decreased from 278 to a low of 59. At the peak of her LFTs, her model end stage liver disease-sodium (MELD-Na) score was 31, which signifies a 19.6% chance of three-month mortality. Again, speaking to the transplant hepatology service, she was still not a candidate for emergent transplant given her appropriate mental status.

After that, her LFTs began down-trending to normal (Table 1). Her renal function remained very poor and she was still requiring intermittent hemodialysis. She was downgraded from the MICU and was transferred to the psychiatric unit for further management. Upon discharge, she was to follow up with our hepatology clinic for further evaluation.

**Table 1:** Trend of LFTs, albumin, platelets, and INR LFTs: liver enzymes; AST: aspartate aminotransferase; ALT: alanine aminotransferase; ALP: alkaline phosphatase; INR: international normalized ratio

Date	Total Bilirubin	AST	ALT	ALP	Albumin	Platelets	INR
Day 1	0.2	25	19	73	4.1	278	1.1
Day 2	0.3	38	25	58	2.6	260	1.2
Day 3	1.8	302	283	85	2.3	148	1.9
Day 4	2	350	383	90	2.3	140	1.5
Day 5	2.7	410	501	90	2.2	90	1.4
Day 6	4.4	> 717	1411	100	2.3	79	1.3
Day 7	5	> 717	1873	135	2.5	59	1.3
Day 8	3.2	662	1417	120	2.6	105	1.2
Day 9	1.6	224	987	126	2.7	113	1.1
Day 10	1.3	126	819	126	2.7	116	0.9
Day 11	1.1	58	542	122	3.1	129	0.9

## Discussion

In this patient, the initial thought process revolved around ischemic shock liver while ibuprofen toxicity was a secondary differential. However, once her LFTs did not begin to downtrend as expected, ibuprofen toxicity quickly became the leading differential. While it is exceedingly rare in general, it is even rarer in this case because it was not accompanied by any systemic signs or symptoms [1]. That being said, nonsteroidal anti-inflammatory drugs (NSAIDs) are some of the most commonly prescribed medications in the world for a variety of ailments, and they have been shown to cause serious liver toxicity, which has made for a heavy financial burden on healthcare systems [2-3].

Overall, when it comes to acute liver injury, iDILI ranks second to acetaminophen toxicity (11% compared to 46%). Only about 10% of those with iDILI will progress to acute liver failure but about 60% of those will require a liver transplant [4]. A drug-induced liver injury is considered to be one of the most severe adverse drug reactions; it is one of the biggest reasons why many drugs don't make it through testing phases and are pulled from the market, even after approval [5]. The severity of the liver injury is typically unpredictable and the main therapies are the withdrawal of the suspected agent along with supportive care (as well as educating the patient to avoid the inciting medication in the future) [4]. With regards to ibuprofen specifically, one recent review concluded that it had a significant risk of acute liver injury and that it was dose-related; this is quite important given that ibuprofen is the most commonly used NSAID in the world [5]. This case report supports that finding, given the enormous dose of ibuprofen that our patient had ingested.

As far as therapy, corticosteroids have not been shown to play a major role in therapy. NAC may be administered, but the most important prognostic factors are not only early detection but also timely evaluation for a liver transplant [4]. It is also vitally important to withdraw the suspected agent and to make sure that when using drugs known to cause a liver injury that they are appropriately dosed. Thankfully, our patient responded well to NAC and recovered from her liver injury without requiring emergent liver transplantation.

## Conclusions

Rapid, yet thorough, history-taking, as well as having an appropriate set of differential diagnoses is life-saving. Had this patient deteriorated further and had we not thought of a rare cause of liver injury (ibuprofen), she may have perished without the benefit of a possibly life-saving liver transplant evaluation.
